# Reduction of emission level in approach signals of greater mouse-eared bats (*Myotis myotis*): No evidence for a closed loop control system for intensity compensation

**DOI:** 10.1371/journal.pone.0194600

**Published:** 2018-03-15

**Authors:** Tobias Budenz, Annette Denzinger, Hans-Ulrich Schnitzler

**Affiliations:** Animal Physiology, Institute for Neurobiology, University of Tübingen, Tübingen, Germany; Università degli Studi di Napoli Federico II, ITALY

## Abstract

Bats lower the emission SPL when approaching a target. The SPL reduction has been explained by intensity compensation which implies that bats adjust the emission SPL to perceive the retuning echoes at the same level. For a better understanding of this control mechanism we recorded the echolocation signals of four *Myotis myotis* with an onboard microphone when foraging in the passive mode for rustling mealworms offered in two feeding dishes with different target strength, and determined the reduction rate for the emission SPL and the increase rate for the SPL of the returning echoes. When approaching the dish with higher target strength bats started the reduction of the emission SPL at a larger reaction distance (1.05 ± 0.21 m) and approached it with a lower reduction rate of 7.2 dB/halving of distance (hd), thus producing a change of echo rate at the ears of + 4 dB/hd. At the weaker target reaction distance was shorter (0.71 ± 0.24 m) and the reduction rate (9.1 dB/hd) was higher, producing a change of echo rate of—1.2 dB/hd. Independent of dish type, bats lowered the emission SPL by about 26 dB on average. In one bat where the echo SPL from both targets could be measured, the reduction of emission SPL was triggered when the echo SPL surpassed a similar threshold value around 41–42 dB. Echo SPL was not adjusted at a constant value indicating that *Myotis myotis* and most likely all other bats do not use a closed loop system for intensity compensation when approaching a target of interest. We propose that bats lower the emission SPL to adjust the SPL of the perceived pulse-echo-pairs to the optimal auditory range for the processing of range information and hypothesize that bats use flow field information not only to control the reduction of the approach speed to the target but also to control the reduction of emission SPL.

## Introduction

During evolution the echolocation systems of the more than 1200 echolocating bat species have been adapted to perform specific echolocation tasks [1–3]. When closing in on a target of interest bats emit approach signals [2,4]. These approach signals and their returning echoes result in a sequence of pulse-echo pairs which contain the relevant information on target position and its nature. The ability to process and evaluate this information is limited if the sound pressure levels of the emitted signals and the returning echoes are outside of the optimal processing range of the bats’ hearing system. The dynamic range of auditory neurons and in psychophysically measured auditory behaviors is far smaller than the rather wide range between the high emission level of echolocation signals and the detection level for weak echoes [5–8].

Bats searching for small targets such as insects have to emit very loud sounds to produce detectable echoes over longer ranges. Emission SPLs around 131 dB (re 0.1 m) were measured for search signals of bats foraging in open space [9,10]. On the other hand reasonable echo detection levels around 20 dB have been discussed [11]. Thus, bats have to deal with very large amplitude ranges while searching for prey. Even if we assume that the SPL at the bats ear is about 5–15 dB below the SPL measured in front of the bat’s mouth due to the directionality of sound emission and of the pinnae, and that the perceived emission SPL is additionally reduced by 12–22 dB due to the middle ear reflex induced by vocalization [12], the range of perceived SPLs would still be around 80 dB. Such high dynamic ranges between loud calls and the just detectable echoes push the auditory system of foraging bats to its limits. After detection bats have to determine the position of a target of interest with high precision especially if they want to catch it, avoid it, or land on it. This may be the reason why bats approaching a target of interest lower the emission level to adjust the SPL of pulse-echo pairs to the optimal processing range of the auditory system.

A decrease in emission SPL during target approach has been reported by many authors and for many species and is most likely a common behavior in all echolocating bats [1,13–29]. The reduction rate was mostly near 6 dB per halving of the distance (dB/hd) with variations in a range between 4–9 dB/hd (summarized in Table 1 in Koblitz et al [27]), and bats started to lower call amplitude at target distances which were larger in landing bats (around 1.5–2 m) than in bats approaching an insect (around 0.6 m).

Several mechanisms have been proposed to explain how bats regulate the echo SPL during an approach so that an optimal processing of the echo information is possible. Kick and Simmons [30] assumed that the time dependent middle ear reflex has the function of an “automatic gain control” mechanism which for some time after signal emission reduces the auditory threshold of incoming echoes by 11 dB/hd and thus compensates for the increase of echo SPL by 12 dB/hd when closing in on point targets such as insects. Kobler et al. [14] found a range dependent decrease of emission SPL by 20–30 dB over a target distances from 2.5–0.2 m in *Pteronotus parnellii* sitting on a pendulum and termed it “intensity compensation”. Hartley and colleagues [15–17] assumed that bats use a “dual component system for stabilization of perceived echo amplitude” by both, lowering the emission SPL and reducing the auditory perception level during the middle ear reflex.

All these proposed mechanisms somehow imply that the echo SPL is regulated by a feedback control system which keeps the echo SPL approximately constant, similar to the Doppler shift compensation system of flutter detecting bats. Doppler shift compensating bats lower the emission frequency to compensate the positive Doppler shifts produced by their own flight movement so that the echoes returning from large stationary targets ahead are kept constant at the reference frequency [31,32]. A comparable closed loop control system for intensity compensation should adjust the emission SPL to keep the echo SPL at the bat’s cochlea constant at a reference value. Such a system would have to compensate for changes in echo SPL caused by the target’s reflecting properties, by atmospheric attenuation and geometric spreading when closing in on the target but also for changes caused by the vocal middle ear reflex which attenuates not only the emitted signal during sound emission but also echoes which return shortly after sound emission [12]. During the approach the dynamic changes in echo SPL due to spherical spreading are rather large and depend on the reflection properties of a target. The echo SPL of a mirror-like target increases by 6 d/hd, of a wire-like target by 9 dB/hd, and of a point target by 12 dB/hd [11]. The influence of atmospheric attenuation is rather small due to the small target distances. A bat closing in on a target from 240 to 7.5 cm encounters five halving steps due to spherical spreading which sum up to a total increase of echo SPL of 30 dB in a mirror-like wall, of 45 dB in a thin landing bar, and of 60 dB at a point target like an insect. Furthermore, target strength (TS) has a strong additional effect on the absolute echo SPL [29]. At the same distance smaller insects produce lower echo SPLs than larger insects or a wall. Further variations of the echo SPL are caused by changes of target position in relation to the directional emission beam and the directionality of the pinnae and by changes of the reflective parts of the target such as wing beat movements [30].

If emission SPL were controlled by a closed loop control system, the different distance dependent increase rates in echo SPL of targets with different reflecting properties should be compensated by corresponding decrease rates of the emission level. Additionally, at loud targets with high target strength the SPL reduction should start at larger target distances than at more quiet targets. These predictions are either not or only partly supported by data from literature. Emission SPL reductions measured so far indicate a rather similar decrease rate around 6 dB/hd independent of bat species and differences in target reflection properties according to target size and type (wall, mealworm, small microphone, spheres). As there is no big difference in the emission level decrease rate between point (12 dB/hd) and mirror (6 dB/hd) targets the published data do not support the negative feedback control hypothesis which requests that emission SPL decrease rate and echo SPL increase rate should compensate each other. Boonman and Jones [19] also rejected a closed loop control system as *Myotis daubentonii* approaching different objects (large and small sphere and mealworm) reacted with a stereotyped SPL reduction by about 4 dB/hd which was independent from the object’s target strength, and [33] argued that strong source level fluctuations of up to 12 dB within sound groups emitted by *Eptesicus fuscus* during an approach do not support the hypothesis of a tightly coupled feedback control system. There is, however, evidence that target strength influences the reaction distance as bats approaching a wall which produces loud echoes started to lower the emission SPL around 2 m from the wall [21,27], whereas bats approaching a mealworm which produces much weaker echoes reacted around 0.6–0.7 m [19,20].

For a better understanding of the mechanisms that regulate the reduction of emission SPL during approach we trained bats to approach two types of feeding dishes with different target strength, and recorded the emitted echolocation signals and, as far as possible, also the returning echoes with an onboard microphone. For both target types we determined the reduction rate of the SPL of the emitted signals during the approach and the resulting increase rate for the echo SPL to test our hypothesis that lowering of emission SPL is not controlled by a closed loop feedback control system for intensity compensation.

## Materials and methods

### Experimental animals

Four males of the Greater mouse-eared bat (*Myotis myotis*, Borkhausen 1974) were used in this study. Bats were captured from a large colony roosting in a cave named ‘Höllenlöcher’ (Dettingen an der Erms, Germany) under the license No. 55-3/8852.21 from March 03, 2009 of the “Regierungspräsidium Tübingen”. Bats were kept under standardized conditions (16:8 light: dark cycle, 24 ± 2°C, and 65 ± 5% humidity) with *ad libitum* access to water. During training and experiment the bats were fed with mealworms (larvae of *Tenebrio molitor*) which were offered in feeding dishes. Bats could fly freely and feed as much as they wanted. The bats’ weight was controlled daily to make sure that they fed enough. Additionally, crickets, beetles and grasshoppers were offered on a regular base. Vitamins (Korvimin^®^, WDT eG, Germany) and fatty acids (Efaderm^®^ liquid, aristavet GmbH & Co, Germany) were supplemented every second week. Every other day the bats were allowed to fly freely for several hours in a large flight room. All experiments were approved by the “Regierungspräsidium Tübingen” (Permit No. 35/9185.81–2, ZP 2/08).

### Experimental setup

The experiments were conducted in the dark in a large flight room (13 x 6 x 2 m). The flight room was separated with a curtain into an experimental and an equipment area. On the floor of the experimental area (7.85 x 6 x 2 m) we positioned a Plexiglas plate (3 x 2 m). On the smooth surface of this feeding area we offered four feeding dishes (r = 4.5 cm, h = 1.5 cm) always at the same positions. One of these dishes contained live mealworms. The bats were trained to search for the dish with mealworms and to approach and catch them after detection. At the beginning of each trial the bat was released at one end of the flight room and was allowed to fly freely in the experimental area and to search for the dish with prey. We used two different sets of feeding dishes which differed in their reflection properties to investigate whether target properties influence the echolocation behavior of bats. The first set consisted of plastic Petri dishes with a corner reflector made of aluminum foil (height 2 cm) to increase the reflectance (reflector dish), the second set comprised similar sized dishes consisting of cellular foam without a reflector (foam dish). The feeding dishes contained small styrofoam pieces which prevented the detection of the mealworms by echolocation and which generated rustling noises when living mealworms moved between them. In each trial we used the same type of feeding dish, but only one dish, the target dish, contained living mealworms. The position of this target dish was changed from trial to trial in a randomized manner between the four dish positions on the Plexiglas plate.

### Data recording and analysis

We recorded the echolocation and flight behavior of the bats with a 3D infra-red video system and synchronized sound recordings. Echolocation signals were recorded by a custom-made telemetry system, the telemike, consisting of a microphone and a transmitter. The bats carried the telemike in a small backpack mounted on the back of the bat. The backpack was fixed with a small collar around the neck of the bat and stabilized with a drop of medical skin adhesive (Sauer GmbH, Lobbach, Germany) on a naked skin mark on the back of the bat. The adhesive and with it the backpack were easy to remove after the experiment without harming the bats. The telemike consisted of a small omnidirectional ultrasonic sensor (Knowles electronics, SPM0204UD5, USA), an FM-transmitter unit with antenna (180 MHz), and a button cell of 1.55 V (Varta, V 377, Hannover, Germany). The weight of the telemike including the battery was 1.5 g, which was light enough to be carried by Greater-Mouse Eared bats weighing about 30 g. Recording sessions lasted not longer than 30 minutes for each bat and recordings were made on five days per week. The telemike signals were picked up by two VHF-antennae which were connected with a custom-made fm-receiver with a carrier frequency range between 174–223 MHz. The transmitted signals were digitized with a custom-made A/D-converter (PC-Tape, Department of Animal Physiology, University of Tübingen) at a sample rate of 480 kHz and a resolution of 16 bits, and stored on a hard disk of a personal computer as wav-files. Sound recordings were synchronized in time with a vertical interval time code (VITC) with video recordings.

The flight behavior of the bats was recorded with two infrared-sensitive cameras (Sanyo IRP, 50 half-frames per second), fixed in two upper corners of the experimental area. The flight room was illuminated by two infrared stroboscopes which were synchronized with the cameras and released a 1 ms long flash at every half-frame. The video data were recorded on Panasonic DVC mini video tapes with two Sony camcorders (DCR-PC8E). An additional camera with a telephoto lens was used to take close-up recordings of bats approaching the target dish. For the 3D reconstruction of the flight paths we digitized the videos and analyzed them frame by frame with commercial software (SIMI Motion^®^ 3D, Version 7.5, SIMI reality motions GMBH, Unterschleißheim, Germany). The reconstruction accuracy was better than 4 cm.

Calibration signals, echolocation signals and echoes were analyzed with a custom-written program (Selena, University of Tübingen, Germany). The sounds were displayed as color spectrograms (FFT 256, Hann window) with a dynamic range of 90 dB and analyzed with auto padding at a window width of 20 ms and a frequency range reduced to 120 kHz. The resulting interpolation process produced an accuracy of 0.5 ms in the time and 0.9 kHz in the frequency domain. The beginning and the end of the signals were determined at -15 dB below peak frequency amplitude and pulse interval, pulse duration, initial and terminal frequency, bandwidth, and SPL were measured. In total, we recorded 243 search flights and 330 approach flights of which we analyzed 3 search flights and 5 approach flights for each type of feeding dishes for each of the four bats.

### Computation of emission and echo SPL

A custom written program called INAT (intensity analysis tool, University of Tübingen, Germany) was used to calculate the root mean square SPL relative to full-scale of the echolocation signals, and the echoes which were recorded with the telemike system. The signals could be pre-processed by a definable band pass filter. All SPL values presented in this paper were measured at a band pass setting between 15–200 kHz.

### Criterion to define the active part of the approach and to calculate SPL reduction rate

The active part of the approach started when the bats reacted to the target with distinct changes in their echolocation behavior. Our criterion for this change was that the SPL of the first call of the final approach had to be 2 dB lower than the mean SPL of the preceding calls and that it was further reduced in the following calls. The 2 dB criterion was chosen as it is equivalent to double standard deviation of the average emission SPL in passive approach.

### Calibration of the telemike

For the calibration of the telemike we used a custom-made ultrasonic loudspeaker which was calibrated with a 1/8” Brüel & Kjær microphone in the frequency range from 15–110 kHz. The telemike had its highest sensitivity at 40 kHz and was 4 dB less sensitive at 20 kHz and 13 dB at 90 kHz. The dynamic range of the complete recording (telemike and PCtape recording system) was determined for a signal frequency of 40 kHz and measured 47 dB. It was limited by the noise level of about 40 dB SPL and a saturation point of the input-output function of about 87 dB.

### Ensonification of the feeding dishes

To determine the acoustic reflection properties of our feeding dishes we used a custom-made ultrasonic loudspeaker which had a rather flat response curve between 15 kHz and 110 kHz with a maximum output at about 60 kHz and a 3 dB lower SPL at 30 kHz and 100 kHz. The dishes were ensonified with bat-like pulses generated with a waveform generator (Agilent 33120A). The pulses had a SPL of 86 dB at 40 cm and swept from 110–30 kHz in 2 ms. The echoes were picked up with a calibrated custom-made microphone which was positioned next to the loudspeaker. For signal recording and analysis, we used the same recording system and analysis software as described above (PC-Tape, Selena, INAT). To determine the distance dependent change in echo SPL we positioned the dish at seven distances in front of the ensonification system (0.4–1 m, steps of 0.1 m). At every distance, the mean echo SPL was determined from eight measurements, where the dish was turned by 360° in eight steps. The dishes were ensonified in two different angles according to the mean bats approach angles (16°, 24°).

### Statistical analysis

The results were quantified using commercial statistic software (SPSS 20, IBM, Chicago, USA). Pulse interval was not normally distributed (Shapiro- Wilk test, p<0.05), therefore, we calculated median and interquartile range, and used Mann-Whitney-U test to compare pulse interval of search flight and passive approach. All other datasets were normally distributed (Shapiro-Wilk test, p>0.05). Therefore, we conducted general linear mixed model (GLMM) analysis with individual bat as random factor to compare (1) the microphone SPL of approaches from the walls vs. approaches from search flights (fixed factor: either approach from wall or search flight); (2) the echolocation signal parameters of search flight and passive approach (fixed factor: search or passive approach); and (3) the bats’ position and approach angles at the beginning of SPL reduction (fixed factor: type of feeding dish). We calculated multiple linear regression analysis to test for the increase in echo SPL during the landing task to the reflector dish, to determine the SPL reduction rates of approaching bats and the increase rates of the ensonification experiment. We used t-tests for dependent variables to compare the ensonified feeding dishes and SPL reduction rates. A one-sample-t-test was used to test whether the mean SPL reduction rate during the landing at the reflector dish differs from the reduction rate 6 dB/hd.

## Results

### Flight and echolocation behavior of *M*. *myotis* while searching for and approaching feeding dishes with prey

**Fig 1:**
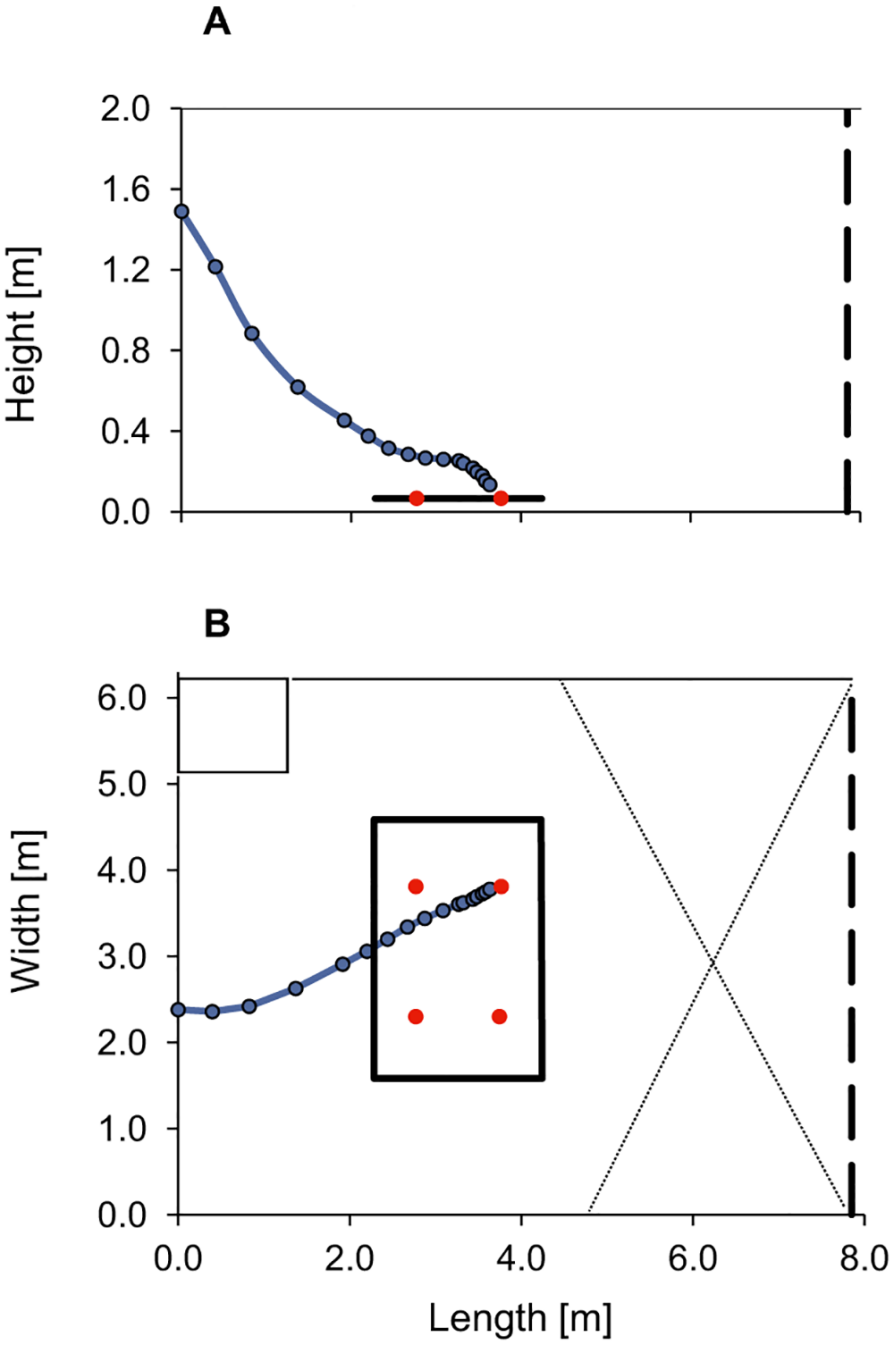
Side (A) and top-view (B) of a typical flight trajectory during an approach from the wall (bat C). The dots indicate the positions where the bat emitted calls during flight. The black marked area represents the feeding area with the four feeding sites. The echolocation behavior during this passive part of the approach was similar whether they approached the dish directly from search flight or from the wall. SPL of signals emitted during passive approach did not differ (GLMM, F_(1, 219)_ = 0.28, p = 0.598). Therefore, we used pooled data from the two behavioral situations to describe the echolocation behavior during the passive approach. However, we did not pool data of individual bats.

For the description of the flight and echolocation behavior of *M*. *myotis* foraging in the passive mode we discriminate three different behavioral stages: search, passive approach and active approach. After releasing the bats, all 4 individuals immediately began to search for prey. They flew in large circles over the feeding area at average heights between 0.6 m and 1.4 m and average speeds between 3.0 m and 4.2 m/s. Rustling sounds of moving mealworms from one of the four familiar feeding dishes at known positions enabled the passive localization of the target dish with prey. When bats had detected the prey generated sound source they switched from search to the passive approach which was indicated by a distinct change in flight behavior. Bats either reduced flight height and flew in a wide circle to the position of the target dish or they landed at the wall at heights of 1.2–1.8 m and flew from there directly towards the dish, which was around 3–4 m away (Fig 1). In both situations, bats started ‒ at a distance to the target of about 2–3 m ‒ to fly in more or less horizontal flight at heights of 0.2 ‒ 0.4 m. This passive acoustically guided approach ended when the bats had picked up the target dish by echolocation and switched to active approach, which is guided by echolocation. The active approach ended, when bats either landed on the dish with prey or close to it during a down stroke of the wings.

**Fig 2:**
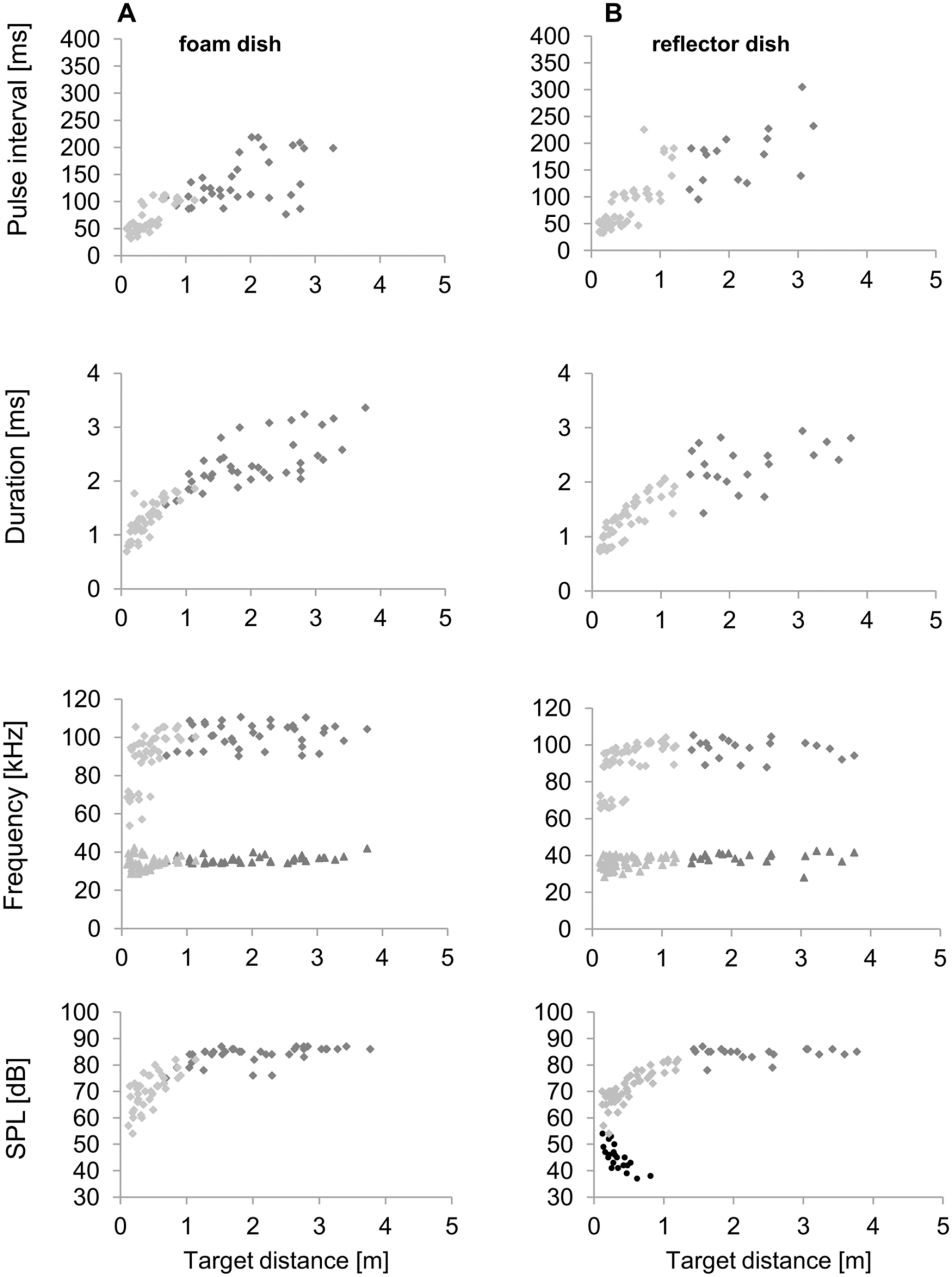
Echolocation parameters for 10 approach flights of bat C (6 approaches from wall, 4 approaches from search flight) when landing on the foam dish (A) and on the reflector dish (B). Dark gray represents search and passive approach, light gray symbols active approach. The black circles indicate the echo SPL of the reflector dish.

The echolocation behavior during passive approach differed from the echolocation behavior in search flight by shorter pulse intervals (Mann-Whitney-U test, U = 40995.5, n = 725, p < 0.001), higher initial frequency (GLMM, F_(1,773)_ = 88.27, p < 0.001), lower terminal frequency (GLMM, F_(1, 775)_ = 5.6, p = 0.018), lower emission SPL (GLMM, F_(1, 768)_ = 20.02, p < 0.001) and shorter pulse durations (GLMM, F_(1, 720)_ = 83.15, p < 0.001) (Table 1 and Fig 2). The higher initial frequency and lower terminal frequency indicate that the bats increased the bandwidth of echolocation signals during the passive approach. Pulse intervals of mainly between 90 ms and 100 ms indicate that the bats emitted a single call every wingbeat. The longer pulse intervals in search flight suggest that bats sometimes emitted signals only every second wing beat when flying higher. The switch from passive to active approach was indicated by a reduction of emission SPL and also of pulse interval and pulse duration (Fig 2). Pulse intervals were reduced to minimal 32 ms and pulse duration to 0.7 ms.

**Table 1:** Signal parameters of the echolocation pulses emitted during search and in passive approach.

	Search phase	Passive approach	P
	(3 search flights per bat)	(10 flights per bat)	
PI (ms)	148.8(104.3–202.7)	104.6(89.8–153.5)	<0.001
	n = 123	n = 291	
IF (kHz)	88.8±9.9	93.1±7.9	<0.001
	n = 448	n = 330	
TF (kHz)	38.2±4.1	36.93±4.6	0.018
	n = 449	n = 331	
PD (ms)	2.6±0.6	2.3±0.5	<0.001
	n = 448	n = 330	
SPL (dB)	85.2±3.4	84.4±2.6	<0.001
	n = 450	n = 322	

PI = pulse interval, IF = initial frequency, TF = terminal frequency, PD = pulse duration, SPL = sound pressure level. Except for PI, values correspond to mean ± SD. The p-values result from linear mixed model analysis with individual bat as a random factor. PI was not normally distributed and thus subject to non-parametric statistics (Median, interquartile range, Mann-Whitney-U test).

During active approach, the bats either continued to emit single pulses before they switched to groups with two, sometimes three, and rarely even 4 pulses—or they started immediately with the emission of grouped signals. In one approach bat D emitted only single pulses. Two bats emitted ‒ rather often ‒ (Bat A: 70% and Bat D 80% of all landings) a series of 3–6 short, broadband signals, with pulse durations around 1.5 ms and intervals around 6 ms, shortly before or during the landing on the dish. The emission SPL was reduced throughout the active approach, but in two bats it increased again in the series of these short pulses emitted just before or during landing on the dish. Sometimes the SPL of the last pulse group before landing or only of the last pulse of this group was also increased. The SPLs of these pulses were not included in describing the SPL reduction during active approach because these louder pulses were emitted less than 60 ms prior to landing, which is shorter than the reaction time.

### Emission and echo SPL during active approach

**Fig 3:**
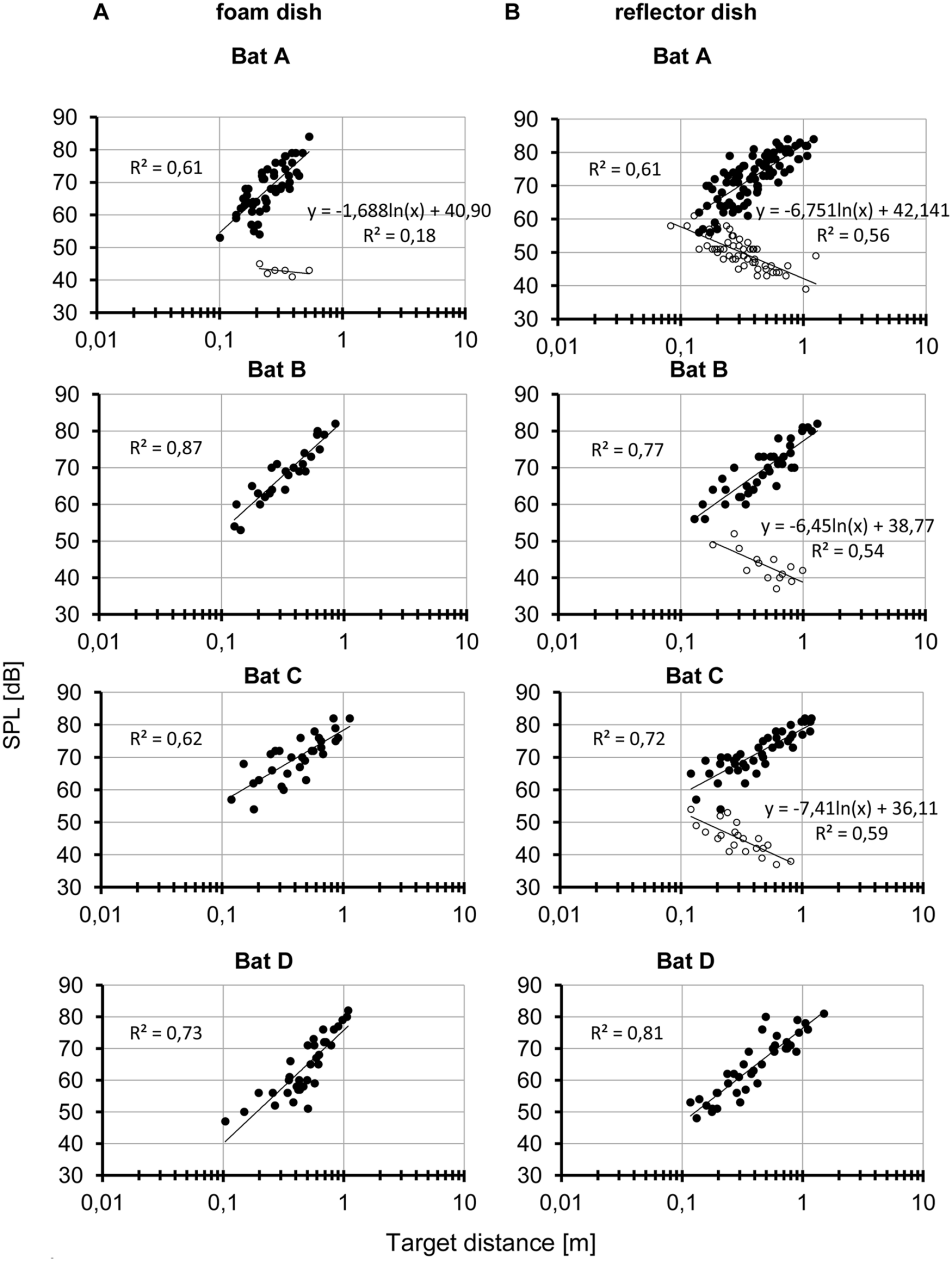
Logarithmic regression analysis of emission SPL reduction (filled circles) and echo SPL increase in relation to target distance (open circles) of all signals emitted during the active approach from 5 landings of each bat on the foam (A) and on the reflector dish (B). Echoes from the foam dish were only recorded during one flight of bat A.

In search and passive approach emission SPL was kept at about 85–86 dB SPL rms on average (Table 2). During active approach, the four bats lowered the SPL of emitted signals when closing in on the target (Fig 3). The mean SPL reduction was 26 dB independently of feeding dish type. Bat D decreased the emission SPL even by 33 dB, thus indicating inter-individual differences. The SPL reduction rates in relation to the logarithm of distance differed in the two target types and were also characterized by large inter-individual variations (Table 3). SPL reduction rates of bats landing at the foam dish were steeper than the reduction rates of bats landing at the reflector dish (mean reduction rate for at the foam dish 9.1 dB/hd for, and 7.2 dB/hd at the reflector dish, paired t-test, p = 0.028). The echoes from the reflector increased by 4.8 dB/hd on average (Fig 3 and Table 3).

**Table 2:** Mean emission SPL in dB (rms) in search and passive approach flight and mean minimal SPL of active approach during all landings (n = 20 for each dish type).

Bat	Mean SPL	Mean SPL	Mean min. SPL	Mean min. SPL	SPL reduction	SPL reduction
	Search and passive approach					
	foam	reflector	foam	reflector	foam	reflector
**A**	84.2	85.8	58.4	58.0	25.8	27.8
	n = 49	n = 41	n = 5	n = 5		
**B**	83.7	84.1	57.9	61.0	25.8	23.1
	n = 52	n = 31	n = 5	n = 5		
**C**	83.8	84.4	62.4	64.6	21.4	19.8
	n = 38	n = 21	n = 5	n = 5		
**D**	83.4	84.6	50.5	51.5	32.9	33.1
	n = 72	n = 41	n = 5	n = 5		
**mean**	83.8	84.8	59.0	60.9	26.5	26.0

The difference between mean and minimal SPL indicates the SPL reduction during active approach.

**Table 3:** SPL reduction rate, increase rate of echo SPL and approach angles (mean ± SD) of the bats.

Bat	Reduction rate	Reduction rate	Increase rate	Approach angle	Approach angle
	foam	reflector	echo SPL	foam	reflector
	dB/hd	dB/hd	dB/hd	[°]	[°]
A	10.2	7.1	4.7	36.1±8.1	14.9±3.6
				n = 5	n = 5
B	9.1	7.1	4.5	21.8±5.4	17.7±2.6
				n = 5	n = 5
C	6.6	5.8	5.1	22.9±6.2	15.2±1.5
				n = 5	n = 5
D	10.6	8.9	-	13.9±2.7	17.9±7.9
				n = 5	n = 5
mean	9.1	7.2	4.8	23.7±9.8	16.4±4.4

Reduction and increase rates are given as dB per halving of distance.

Due to technical limitations, the telemike allowed only direct echo SPL measurements above about 40 dB. To determine the echo SPL at the beginning of the active approach we used the measured echo increase rate to predict the average echo SPL at the average target reaction distance. For the reflector dish, we calculated an echo SPL of 42 dB in Bat A, 39 dB in Bat B, and 36 dB in Bat C. For the foam dish, we got only values from Bat A and determined a reaction threshold of 41 dB.

### Position of the bats at the beginning of active approach and approach angle

**Fig 4:**
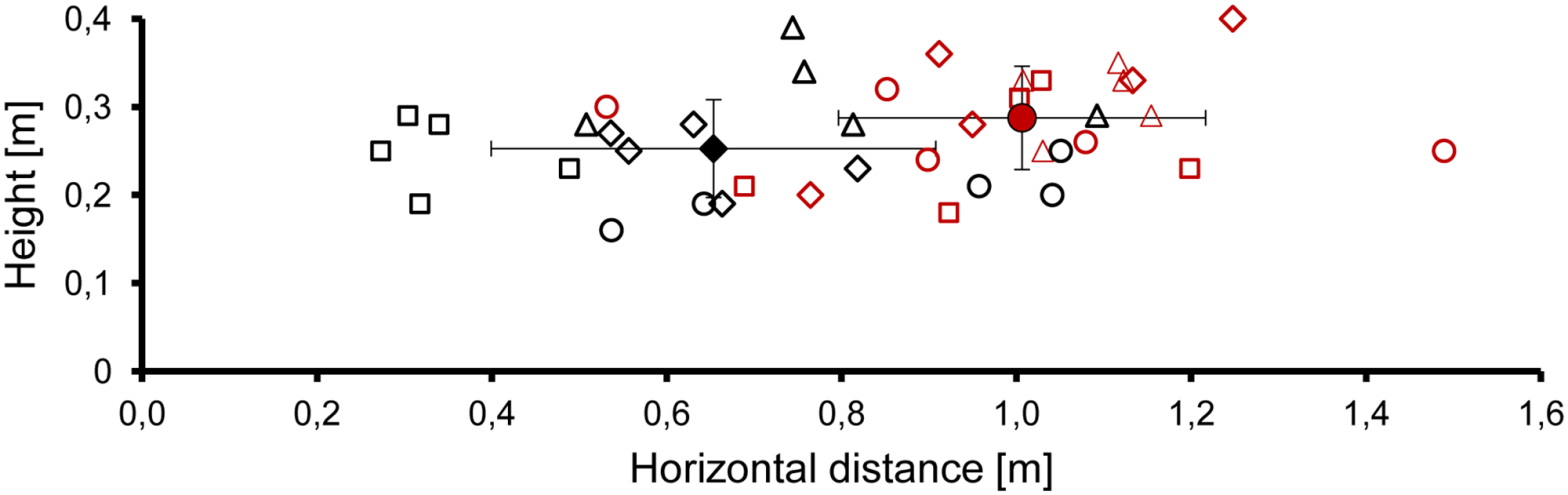
Individual positions of all bats at the beginning of SPL reduction in 5 active approaches to the foam dish (black symbols) and 5 active approaches to the reflector dish (red symbols). Mean positions for all bats are marked with filled symbols (mean ± SD). Squares: bat A, diamonds: bat B, triangles: bat C, circles: bat D.

When approaching the reflector dish bats started to reduce the SPL at a greater horizontal target distance than during the approach of the foam dish (mean ± SD, reflector dish 1.01 ± 0.21 m, and foam dish 0.65 ± 0.25 m, GLMM, F_(1, 35)_ = 27.25, p < 0.001, Fig 4). The average direct target distance at the beginning of the SPL reduction also differed between dish types (mean ± SD, reflector dish 1.05 ± 0.21 m, and foam dish 0.71 ± 0.24 m, GLMM, F_(1, 35)_ = 28.27, p < 0.001). The mean flight height at the beginning of the SPL reduction differed between feeding dish types (mean ± SD, 0.25 ± 0.06 m at the foam dish, 0.29 ± 0.06 m at the reflector dish, GLMM, F_(1, 35)_ = 4.75, p = 0.036).

Bats that landed at the foam dish had in the average a steeper approach angle compared to landings on the reflector dish (mean ± SD, 23.7° ± 9.8 for the foam dish, 16.4° ± 4.4 for the reflector dish, GLMM, F_(1, 35)_ = 10.65, p = 0.002, Table 3).

### Increase rate of echo SPL

**Fig 5:**
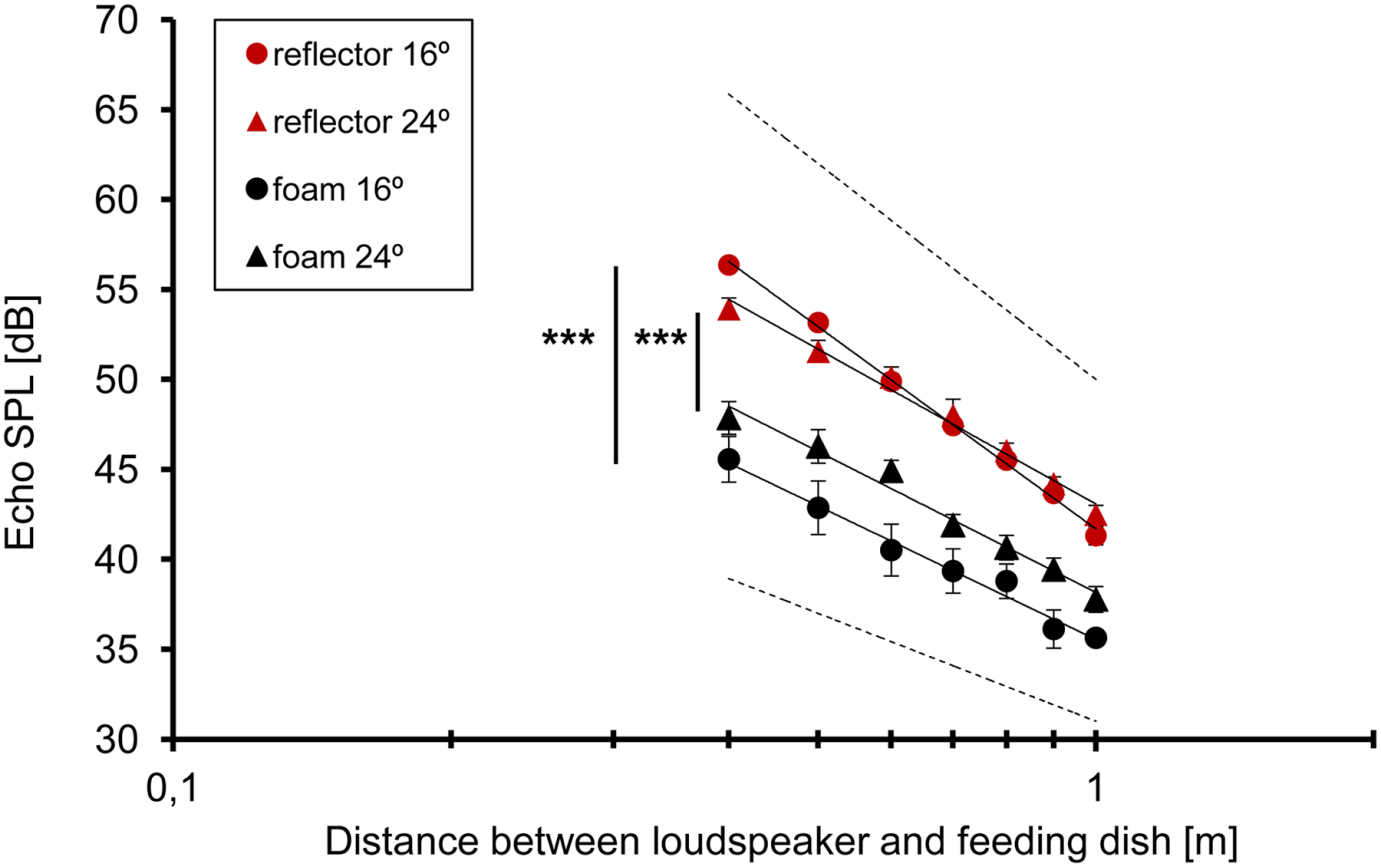
Distance related increase in echo SPL (mean ± SD) of the two types of feeding dishes measured in an ensonification experiment with a signal sweeping in 2 ms from 110–30 kHz with a SPL of 86 dB at 0.4 m. The dashed reference lines indicate slopes of 12 and 6 dB/hd. Asterisks indicate statistical differences in echo SPL. (Regression equations: reflector 16° y = -37.3log(x) + 41.7; reflector 24° y = -28.6log(x) + 43.1; foam 16° y = -24.6log(x) + 35.5; foam 24° y = -26.0log(x) + 38.2).

To determine the distance dependent increase rate of the SPL of the two targets, we ensonified the two feeding dishes at the bats’ approach angle of 16° and 24° (Fig 5) and compared it with the increase rate measured from the echoes in the different experimental situations. When simulating an approach to the reflector target with an ensonification of 16° the echoes increased with a rate of 11.2 dB/hd whereas at a simulated approach to the foam target with 24° the increase rate was at 7.9 dB/hd. In bats where we could measure the SPL of the returning echoes the difference between echo SPL and emission SPL corresponded to the increase rate of the echoes. The measured 10.8 dB/hd corresponded closely with the value of 11.2 dB/hd measured with the loudspeaker at the same angle.

## Discussion

### Echolocation behavior of *Myotis myotis* while foraging in the passive gleaning mode

*Myotis myotis* belongs to the guild of narrow space passive gleaning foragers and uses prey-generated cues to detect and classify prey and to localize its position. Gleaners approaching a site with prey reduce pulse interval and duration but do not emit a distinct terminal group or buzz like aerial-hawking and or trawling bats [2,3,34,35]. In our experiments, the rustling noises of mealworms from the prey dish enabled *M*. *myotis* to detect the prey indicating sound source by passive acoustic localization. After detection bats switched from search to approach behavior, which we divided in two consecutive parts: passive and active approach. During passive approach, the bats are guided by the acoustic cues from the sound source. In this part of the approach *M*. *myotis* lowered flight height and flew in a more or less horizontal flight towards the prey dish at heights between 0.2–0.4 m. The parameters of the echolocation signals emitted during search and passive approach differed only slightly which can be explained by differences in flight height. During active approach, the bats’ flight is guided by echolocation and the signal parameters pulse interval, signal duration, and emission SPL is reduced with decreasing distance to the prey dish. Russo et al. [35] recorded approach signals with one microphone and used the measured changes of signal SPL to define 4 consecutive phases in passive gleaning *M*. *myotis* and *M*. *blythii*. We assume that their phase 1 describes search and passive approach in which signal SPL is high and varies little. The following phase 2 and 3 describe the behavior which we defined as active approach and in which pulse interval, pulse duration and emission SPL is reduced. Our bats did not produce a terminal buzz as observed in phase 4 by Russo et al. [35]. Two bats produced a few short and often louder signals just before or during landing. According to our opinion these signals were irrelevant in guiding the approach as the time to landing was shorter than the reaction time of bats. Russo et al. [35] and Arlettaz et al. [36] concluded that gleaning *Myotis* lower the emission SPL during the approach to reduce the risk of masking prey generated sounds during passive listening, or to avoid alerting ultrasound-sensitive prey. In the following discussion, we argue that *M*. *myotis* reduce the emission SPL during target approach to adjust the SPL of the perceived pulse-echo-pairs to the optimal auditory range for the processing of range information. However, this does not exclude that the lowering of the emission SPL improves the passive detection of prey generated sounds as proposed by Russo et al. [35] and Arlettaz et al. [36].

*M*. *myotis* is a passive gleaning forager and combines passive listening for prey detection and active echolocation for spatial orientation and landing control. This poses the question whether the observed reduction of emission SPL during approach flights can be generalized to other species with different foraging behavior. Aerial-hawking, trawling, and flutter detecting bats also lower the emission SPL when approaching targets under echolocation control [1,13–29] although they do not listen to prey-generated sounds. We therefore assume that our conclusion from data of *M*. *myotis* also applies for other species.

### Detection threshold and rate of change in emission and echo SPL during active approach

Active approach started, according to our definition, when the bats had detected the target dish and started to reduce the emission SPL. The mean reaction distance and the rate of change in emission SPL differed between the two target types. When approaching the louder reflector target the bats reacted at a larger reaction distance (1.05 ± 0.21 m) and with a lower reduction rate (7.2 dB/hd), resulting in a calculated echo change rate of + 4 dB/hd near the ears (difference between echo increase rate of 11.2 dB/hd and emission SPL reduction rate of 7.2 dB/hd). This value was verified by actual measurements of the SPL of target echoes which increased by 4.8 dB/hd. In approaches of the weak foam target reaction distance was shorter (0.71 ± 0.24 m) and the reduction rate (9.1 dB/hd) higher, which led to a calculated echo change rate at the bat’s ears of -1.2 dB/hd. Independent of target reflection properties bats lowered the emission SPL by about 26 dB on average.

In bat A, the telemike recordings allowed to determine the SPL of target echoes from the reflector and the foam target. In this individual, the SPL reduction of the approach signals started in both target types at about the same level of the target echoes namely at about 42 dB (reflector target) and 41 dB (foam target). This result suggests that the reduction of emission SPL is triggered when the echo SPL surpasses a threshold value which was around 41–42 dB. The correlation curves of the reflector echoes from bat B and C predict somewhat lower detection thresholds around 39 dB in bat B and around 36 dB in bat C. This low value explains why we could not record the foam target echoes with the telemike which had a noise level around 40 dB. For further discussion of emission and detection thresholds we will use the values of bat A with an emission level around 85 dB and a detection level for both target types around 41 dB.

A detection threshold of 41 dB seems to be rather high, but in the presence of loud clutter, such as the vertical ground echo returning just before the dish echo, a masked detection threshold is reasonable and corresponds to high thresholds found in other species under masking conditions [37]. We therefore assume that the beginning of active approach indicates the bats had detected the dishes immediately before and had switched from passive target localization to active localization by echolocation. During passive approach, the bats have not yet detected the target dish by echolocation and were searching for it by emitting echolocation signals toward the assumed target position. The differences in echolocation behavior during search and passive approach can mainly be explained by the differences in flight height.

### Perceived emission and echo SPL during active approach

In our experiments, we determined the SPL of the emitted signals and if possible also of the returning echoes with a microphone which was positioned near the bats’ ears. We assume that the SPLs of the telemike signals are a good approximation of the SPLs of the signals which were picked up by the pinnae of the bats. However, for the understanding of our data it is important to remember that the perceived echo SPL at the cochlea–which is the relevant input into the auditory system—is lower than the SPL at the pinna and much lower than the SPL measured directly in front of the bat. In vocalizing bats, the pinna input is attenuated by the middle-ear muscles reflex (vocal MEM reflex) which occurs during sound emission. Hartridge [38] previously hypothesized that the middle ear muscles of bats contract during sound emission and relax afterwards to protect the ear from the loud echolocation signals and to make it sensitive for the perception of weak echoes. Henson [12] recorded cochlear microphonics from vocalizing *Tadarida brasiliensis* and found that the vocal MEM reflex began 4–10 ms before sound emission, had a maximum attenuation effect of about 20 dB at the onset of a signal and ended within 10 ms. If we assume a similar MEM reflex activity in *M*. *myotis* the 85 dB of the telemike signals during the passive approach would result in a cochlear input or perceived SPL of 65 dB. The vocal MEM reflex also attenuates the SPL of echoes if they return shortly after sound emission—but conclusive data are rare. Patheiger [39] studied in *Eptesicus fuscus* how the vocal MEM reflex affects the detection threshold for a wire target offered at different distances, and found at emission levels of 100–105 dB that the detection threshold stayed about the same beyond 90 cm which corresponds to an echo delay of more than 5.3 ms. At shorter distances, the threshold increase was about 4 dB at 60 cm distance and about 11 dB at 30 cm. The curve of the measured threshold increase showed no linear relationship between the detection threshold measured in dB and the logarithm of target range.

Furthermore, no data is available that shows whether the MEM reflex of bats changes with the emission SPL. We do know from EMG measurements in humans [40] that a reduction of emission SPL from 100 dB to 70 dB resulted in a strong decrease of middle ear muscle activity which should result in a reduction of the attenuation effect. We therefore assume a similar mechanism in bats and hypothesize that the reduction of emission SPL during an approach reduces or even abolishes the attenuation effect of the MEM reflex.

During active approach, the relationship between perceived emission and echo SPL is changed due to the reduction of emission SPL, the middle ear reflex, and the distance dependent increase of echo SPL. At the beginning of active approach, the MEM reflex reduces the cochlear input by 20 dB so that the perceived emission SPL of the bats would be at about 65 dB for both target types. The reaction threshold of 41 dB in bat A was about the same at both target types. At the measured reaction distances (1.05 m and 0.71 m) the MEM reflex has no‒or only a minor‒effect on the echo SPL, so that bat A would have perceived pulse echo pairs with a SPL dB relation somewhere near 65:41 at the beginning of the active approach. At 10 cm distance to the dishes the emission level was about 26 dB lower at both targets. If we assume that the effect of the MEM reflex is no more active at the reduced emission level the perceived emission level of bat A would correspond to the SPL of the emitted signal. That means that bat A would hear its own signals at both target types somewhere near 59 dB. At this distance, the calculated echo levels of the two targets types differed according to their different echo change rates. The reflector target which increases with 4 dB/hd would produce echo levels around 55 dB and the foam target which changes with -1.2 dB/hd would produce levels around 38 dB. Measured echo levels support this estimation for the reflector target (Fig 3). By reducing the emission SPL bat A adjusted the pulse-echo SPL relation of the reflector target from 65:41 dB at 1.05 m to 59:55 dB at 0.1 m and at the foam target from 65:41 at the reaction distance of 0.65 m to 59:38 dB at 0.1 m.

### The optimal SPL range of the bats’ auditory system for the processing of range information in perceived pulse-echo-pairs

The accuracy of parameter measurements in echolocating bats depends strongly on the absolute SPL and the relative SPL relation of pulse echo pairs. In *E*. *fuscus* performing a range discrimination task with phantom targets (S+ at 0.53 m and echo SPLs varying between -8 to -48 dB relative to the emission level at 13 cm in front of the bat) the ranging accuracy depended on the relation between emission and echo SPL [7,8]. Performance was best if the echo SPL at the bats’ ears was -28 dB relative to the emission level of about 90 dB and deteriorated at higher and lower echo SPLs. For the calculation of the perceived emission SPL we use data from Henson who showed for *Tadarida brasiliensis* that the own emitted signals with an emission level of about 100 dB (measured a few cm in front of the bat) produced microphonic potentials with the same amplitude as those produced by a tonal signal with an SPL of 65–75 dB [12]. He accounted the difference to the attenuation effect of the middle ear muscles during vocalization by about 20 dB and to the directionality of sound emission which resulted in a lowering of the SPL at the ear by about 5–15 dB. Under the assumption of a similar attenuation effect of about -28 dB through directional sound emission (-10 dB) and MEM effect (-18 dB) *E*. *fuscus* would have reached the best ranging performance if perceived emission and echo SPL were about the same, at an estimated SPL relation of the perceived pulse-echo-pairs of about 62:62 dB.

The importance of the absolute and relative SPL of pulse-echo pairs for the ranging performance of bats is also documented by studies which investigated the neural ranging mechanisms of bats which use broadband frequency-modulated signals. In these experiments the bats were stimulated with a loud signal which mimics the emitted pulse followed by a weak signal which mimics the echo [5,6]. In *Myotis lucifugus* cortical neurons with a so-called paradoxical latency shift reacted to strong signals with a longer response latency than to weaker stimuli and the difference between the two corresponded to the best delay of the neuron. These shifts only occurred when the stimulus amplitude range for the weak stimuli had an upper limit from 29–53 dB SPL and for strong stimuli a lower limit from 62–84 dB SPL. This means that this ranging mechanism only works if the SPL of emitted signals is above 62 dB and of the SPL of returning echoes at least below 53 dB [5]. When probing the properties of echo delay coding neurons in the cortex of 3 bat species with simulated pulse-echo-pairs Hechevarria et al. [41] showed that the absolute and relative SPL of pulse-echo pairs is relevant for the ranging performance of bats. At a simulated emission level of 70 dB the neurons had the highest activity at echo levels between 50–70 dB.

Both the psychophysical data in *E*. *fuscus* [7], and the electrophysiological data from cortical delay tuned neurons of several bat species [5,41], indicate a rather limited operational range for range measurements with a best performance at perceived emission SPLs near 80–60 dB and echo SPLs from 60–40 dB. We therefore conclude that the reduction of emission SPL during the final part of the approach has the advantage of adjusting the SPL of pulse-echo-pairs to the optimal range of the auditory system for processing range information. This conclusion is also supported by behavioral data of Hiryu et al. [21], which allows the estimation of SPL relations of the perceived emission and echo SPL of pulse-echo pairs of *Pipistrellus abramus* landing at a wall. If we assume that the emission SPL measured with the telemike (102 dB) corresponds to the SPL of the signals at the pinna and that the MEM reflex attenuates with 20 dB the perceived emission level at the cochlea would be at 82 dB. The bat started the approach at a distance of about 2 m from the wall. At that point, the echo level measured with the telemike was about 40 dB below the emission level. As the MEM reflex will not influence the perceived echo SPL at a target distance of 2 m a perceived echo SPL of about 62 dB can be estimated. That means that at the beginning of the approach the bat perceived pulse echo pairs with a SPL ratio of 82:62 dB. At a distance of 12.5 cm his ratio would have changed to 72:62 dB if we assume a reduction rate of 6 dB/hd and a continuous reduction of the MEM reflex during the approach. This example shows that the reduction of emission SPL in landing pipistrelle bats adjusted the SPL of the pulse-echo-pairs to the optimal range for the processing of range information. This also holds for the loud reflector target where the reduction of emission SPL adjusted the pulse-echo SPL relation of the reflector target from 65:41 dB at 1.05 m to 59:55 dB at 0.1 m. In the quieter foam target where the SPL relation was changed from 65:41 at the reaction distance of 0.65 m to 59:38 dB at 0.1 m, the reduction of emission SPL did not improve the ranging conditions but is still in a range were ranging is possible.

### Mechanisms for the adjustment of emission SPL during active approach

Proposed mechanisms such as ‘automatic gain control’ [30], ‘intensity compensation’ [14,21], and ‘dual component system for stabilization of perceived echo amplitude’ [16,17] somehow imply that the reduction of emission SPL and/or the effect of the MEM during and short after sound emission regulate the echo SPL in a closed loop control system in a similar way to Doppler shift compensating bats that keep the echo frequency constant [31,32]. Such a tightly coupled closed loop control system for intensity control would suggest that the perceived echo SPL is kept constant at a reference level. The differences in echo change rates and adjusted echo SPLs reject these intensity compensation hypotheses. This result is supported by other studies where bats did not compensate for differences in echo SPL according to different target strengths [19]. But what are the alternatives? In the literature, and also in this paper, the amplitude adjustment of the pulse-echo pairs during target approach is described with the parameter dB/hd. This is a spatial and not a temporal domain parameter which results from two time dependent behaviors of the bat: the braking or deceleration behavior when slowing down before landing or catching prey, and the change of emission SPL during the approach. Distance-dependent reduction rates of emission SPL near 6 dB/hd, that have been reported for several bat species while approaching so different target types like walls and mealworms, might be advantageous for bats. Large targets have the reflection properties of an acoustic mirror which results in a distance-dependent increase of the echo SPL by 6 dB/hd. A reduction of emission SPL by 6 dB/hd compensates for the echo increase rate completely, such that the echo SPL is kept constant at the target and also at the bat’s ear where it is 6 dB lower than at the target [27]. Such a situation was documented where *P*. *abramus* landed on a mirror-like wall [21]. The bats lowered the emission SPL by 6.5 dB/hd during the 2 m long approach and the SPL was nearly constant in the echoes recorded with a telemike. In other species similar decrease rates were measured when approaching a large mirror-like target, for instance approximately 6 dB in *Pteronotus parnellii* approaching a wall while sitting on a pendulum [14], and 4–9 dB/hd in *E*. *fuscus* landing in front of a wall [27]. Small targets such as mealworms or small microphones have the reflection properties of a point target so that the echo increase rate is 12 dB/hd. This increase rate is only partly compensated by lowering the emission SPL with 6 dB/hd so that the echo SPL at the bats’ ears increases with 6 dB/hd. Such a situation was encountered by *Noctilio leporinus* while approaching a mealworm [15], and by *E*. *fuscus* when a mealworm was moved towards it [17] or when it approached a small microphone [20]. Thus, a distance-dependent lowering of the emission SPL would have the advantage that the rising of the echo SPL at the bats ear would provide information on the nature of the reflecting target. A rise by 6 dB/hd indicates a point target, by 3 dB/hd a wire like target and by 0 dB/hd a mirror-like target.

A stereotyped and complete uncontrolled reduction of emission SPL and approach speed according to an open loop control system where the control action is independent of the controlled process output is rather unlikely. Geberl et al. [42] showed in an elegant approach that aerial-hawking and trawling *Myotis daubentonii* responded to the sudden removal of the prey target with a delayed abortion of relevant parts of their sensory-motor behavior, thus indicating that the approach is under feedback control; Amichai and Yovel [29] showed that landing *Pipistrellus kuhlii* adapt their approach behavior to target distance thus indicating distance-dependent sensorimotor feedback control. The feedback control system for the adjustment of the approach behavior is rather slow. Geberl et al. [42] estimated reaction times around 80 ms. Even vocal reaction times of only 40–60 ms as measured in foraging bats (Denzinger and Schnitzler, unpublished data) exclude fast feedback reactions. According to data from Koblitz et al. [27] we assume that it will last as long as about one sound group or wing beat until bats adjust their echolocation behavior to incoming new information from the preceding sound group.

It is not known what kind of feedback mechanism bats use to adjust the distance-dependent reduction of emission SPL at about 6 dB/hd. Lee and his coworkers [43,44] showed that bats use acoustic flow field parameters to control the approach speed while braking to land with speed 0 m/s, and also while catching a mealworm in a controlled collision. Kugler et al. [45] and Warnecke et al. [46] provided evidence that echo-acoustic flow affects flight in bats, and Bartenstein et al. [47], Beetz et al. [48], and Greiter and Firzlaff [49] described neurons in the auditory cortex of bats that selectively respond to echo-acoustic flow fields. We assume that bats use flow field parameters, e.g., the flow of distance reduction, not only for the control of approach speed but also for feedback control of emission SPL. To answer these questions, it will be necessary to study the relation between 3D positions of bat and prey, the approach speed and deceleration, and the SPL reduction in bats that land on mirror-like targets or capture stationary and moving point target like prey under rather natural conditions. In particular, the results from bats catching moving insects might help to understand potential mechanisms for feedback control with echo-acoustic flow parameters.

## References

[1] Griffin DR. Listening in the Dark. 1^st^ ed New Haven: Yale University Press; 1958.

[2] Schnitzler HU, Kalko EKV. Echolocation by Insect-Eating Bats. BioScience 2001;51(7): 557–569.

[3] Schnitzler HU, Moss CF, Denzinger A. From spatial orientation to food acquisition in echolocating bats. Trends Ecol Evol 2003;18(8): 386–394.

[4] Kalko E, Schnitzler HU. How echolocating bats approach and acquire food In: Kunz TH, Racey PA, editors. Bats: phylogeny, morphology, echolocation, and conservation biology. Washington, DC: Smithsonian Institution Press; 1998 pp. 197–204.

[5] Berkowitz A, Suga N. Neural mechanisms of ranging are different in two species of bats. Hear Res 1989;41(2–3): 255–264. 2808154 10.1016/0378-5955(89)90017-8

[6] Sullivan WE. Possible neural mechanisms of target distance coding in auditory system of the echolocating bat *Myotis lucifugus*. J Neurophysiol 1982;48 (4): 1033 doi: 10.1152/jn.1982.48.4.1033 7143031 10.1152/jn.1982.48.4.1033

[7] Denzinger A, Schnitzler HU. Echo SPL influences the ranging performance of the big brown bat, *Eptesicus fuscus*. J Comp Physiol A 1994;175(5): 563–571. 7965921 10.1007/BF00199477

[8] Denzinger A, Schnitzler HU. Echo SPL, training experience, and experimental procedure influence the ranging performance in the big brown bat, *Eptesicus fuscus*. J Comp Physiol A 1998;183(2): 213–224. 9693992 10.1007/s003590050249

[9] Holderied MW, von Helversen O. Echolocation range and wingbeat period match in aerial-hawking bats. Proc R Soc Lond B Biol Sci 2003;270 (1530): 2293–9. 10.1098/rspb.2003.2487 PMC1691500 14613617

[10] Surlykke A, Kalko EKV (2008) Echolocating Bats Cry Out Loud to Detect Their Prey. PLoS ONE 2008;3(4): e2036 doi: 10.1371/journal.pone.0002036 18446226 10.1371/journal.pone.0002036 PMC2323577

[11] Stilz WP, Schnitzler HU (2012) Estimation of the acoustic range of bat echolocation for extended targets. J Acoust Soc Am 2012;132(3): 1765–1775. doi: 10.1121/1.4733537 22978903 10.1121/1.4733537

[12] Henson OW. The activity and function of the middle-ear muscles in echo-locating bats. J Physiol 1965;180(4): 871–887. 5880367 10.1113/jphysiol.1965.sp007737 PMC1357428

[13] Jen PHS, Kamada T. Analysis of orientation signals emitted by the CF-FM bat, *Pteronotus p*. *parnellii* and the FM bat, *Eptesicus fuscus* during avoidance of moving and stationary obstacles. J Comp Physiol A 1982;148(3): 389–398.

[14] Kobler JB, Wilson BS, Henson OW Jr, Bishop AL. Echo intensity compensation by echolocating bats. Hear Res 1985;20(2): 99–108. 4086383 10.1016/0378-5955(85)90161-3

[15] Hartley DJ, Campbell KA, Suthers RA. The acoustic behavior of the fish-catching bat, *Noctilio leporinus*, during prey capture. J Acoust Soc Am 1989;86(1): 8–27.

[16] Hartley DJ. Stabilization of perceived echo amplitudes in echolocating bats. I. Echo detection and automatic gain control in the big brown bat, *Eptesicus fuscus*, and the fishing bat, *Noctilio leporinus*. J Acoust Soc Am 1992;91(2): 1120–1132. 1313464 10.1121/1.402639

[17] Hartley DJ. Stabilization of perceived echo amplitudes in echolocating bats. II. The acoustic behavior of the big brown bat, *Eptesicus fuscus*, when tracking moving prey. J Acoust Soc Am 1992;91(2): 1133–1149. 1556313 10.1121/1.402640

[18] Tian B, Schnitzler HU. Echolocation signals of the Greater Horseshoe bat (*Rhinolophus ferrumequinum*) in transfer flight and during landing. J Acoust Soc Am 1997;101(4): 2347–2364. 9104033 10.1121/1.418272

[19] Boonman A, Jones G. Intensity control during target approach in echolocating bats; stereotypical sensori-motor behaviour in Daubenton’s bats, *Myotis daubentonii*. J Exp Biol 2002;205(18): 2865–2874. 12177150 10.1242/jeb.205.18.2865

[20] Saillant PA, Simmons JA, Bouffard FH, Lee DN, Dear SP. Biosonar signals impinging on the target during interception by big brown bats, *Eptesicus fuscus*. J Acoust Soc Am 2007;121(5): 3001–3010. 17550198 10.1121/1.2714920

[21] Hiryu S, Hagino T, Riquimaroux H, Watanabe Y. Echo-intensity compensation in echolocating bats (*Pipistrellus abramus*) during flight measured by a telemetry microphone. J Acoust Soc Am 2007;121(3): 1749–1757. 17407911 10.1121/1.2431337

[22] Melcon ML, Denzinger A, Schnitzler HU. Aerial hawking and landing: approach behaviour in Natterer’s bats, *Myotis nattereri* (Kuhl 1818). J Exp Biol 2007;210(24): 4457–4464. 18055634 10.1242/jeb.007435

[23] Hiryu S, Shiori Y, Hosokawa T, Riquimaroux H, Watanabe Y. On-board telemetry of emitted sounds from free-flying bats. Compensation for velocity and distance stabilizes echo frequency and amplitude. 2008;194(9):841–51. doi: 10.1007/s00359-008-0355-x 18663454 10.1007/s00359-008-0355-x

[24] Melcon ML, Schnitzler HU, Denzinger A. Variability of the approach phase of landing echolocating Greater Mouse-eared bats. J Comp Physiol A—Neuroethol Sens Neural Behav Physiol 2009;195 (1): 69–77. doi: 10.1007/s00359-008-0383-6 18998148 10.1007/s00359-008-0383-6

[25] Brinklov S, Kalko EKV, Surlykke A. Intense echolocation calls from two `whispering’ bats, Artibeus jamaicensis and Macrophyllum macrophyllum (Phyllostomidae). J Exp Biol 2009;212(1): 11–20. 19088206 10.1242/jeb.023226

[26] Brinklov S, Kalko EKV, Surlykke A. Dynamic adjustment of biosonar intensity to habitat clutter in the bat Macrophyllum macrophyllum (Phyllostomidae). Behav Ecol Sociobiol 2010;64(11): 1867–1874.

[27] Koblitz JC, Stilz P, Pflaesterer W, Melcón ML, Schnitzler H-U. Source level reduction and sonar beam aiming in landing big brown bats (*Eptesicus fuscus*). J Acoust Soc Am 2011;130(5): 3090–3099. doi: 10.1121/1.3628345 22087937 10.1121/1.3628345

[28] Norum U, Brinklov S, Surlykke A. New model for gain control of signal intensity to object distance in echolocating bats. J Exp Biol 2012;215 (17): 3045–3054. 22875770 10.1242/jeb.069427

[29] Amichai E., Yovel Y. Bats pre-adapt sensory acquisition according to target distance prior to takeoff even in the presence of closer background objects. Sci Rep 2017;7: 467 doi: 10.1038/s41598-017-00543-8 28352130 10.1038/s41598-017-00543-8 PMC5428694

[30] Kick SA, Simmons JA. Automatic gain control in the bat’s sonar receiver and the neuroethology of echolocation. J Neurosci 1984;4(11): 2725–2737. 6502201 10.1523/JNEUROSCI.04-11-02725.1984 PMC6564721

[31] Schnitzler HU. Kompensation von Dopplereffekten bei Hufeisen-Fledermäusen. Naturwissenschaften 1967;54(19): 523. 5586165 10.1007/BF01129387

[32] Schnitzler HU, Denzinger A. Auditory fovea and Doppler shift compensation. adaptations for flutter detection in echolocating bats using CF-FM signals. J Comp Physiol A Neuroethol Sens Neural Behav Physiol 2011;197(5): 541–559. doi: 10.1007/s00359-010-0569-6 20857119 10.1007/s00359-010-0569-6

[33] Koblitz JC, Stilz P, Schnitzler HU. Source levels of echolocation signals vary in correlation with wingbeat cycle in landing big brown bats (*Eptesicus fuscus*). J Exp Biol 2010;213(19): 3263–3268. 20833918 10.1242/jeb.045450

[34] Deutschmann K. Verhalten von M. myotis beim Fang fliegender Insekten und der Lokalisation von Beute auf dem Boden. Diploma thesis, Faculty of Science, University of Tübingen, Germany. 1991.

[35] Russo D, Jones G, Arlettaz R. Echolocation and passive listening by foraging mouse-eared bats *Myotis myotis* and *M*. *blythii*. J Exp Biol 2007;210(1): 166–176. 17170159 10.1242/jeb.02644

[36] Arlettaz R, Jones G, Racey PA (2001) Effect of acoustic clutter on prey detection by bats. Nature 414 (6865): 742–745. doi: 10.1038/414742a 11742397 10.1038/414742a

[37] Møhl B. Target Detection by Echolocating Bats In: Nachtigall PE, Moore PWB, editors. Animal Sonar: Processes and Performance. Boston, MA: Springer; 1988 pp. 435–450.

[38] Hartridge H. Acoustic control in the flight of bats. Nature 1945;156(3965): 490–494. 10.1038/156692b0 21007621

[39] Patheiger S. Die Detektionsschwelle von Eptesicus fuscus bei verschiedenen Zielentfernungen. Diploma thesis, University of Tübingen, Germany. 1998.

[40] Borg E, Zakrisson JE. The activity of the stapedius muscle in man during vocalization. Acta Otolaryngol 1975;79(5–6): 325–333. 1155042 10.3109/00016487509124694

[41] Hechavarría JC, Macías S, Vater M, Voss C, Mora EC, Kössl M. Blurry topography for precise target-distance computations in the auditory cortex of echolocating bats. Nat Commun 2013;4: 2587 doi: 10.1038/ncomms3587 24107903 10.1038/ncomms3587

[42] Geberl C, Brinkløv S, Wiegrebe L, Surlykke A. Fast sensory–motor reactions in echolocating bats to sudden changes during the final buzz and prey intercept. Proc Nat Acad Sci 2015;112(13): 4122–4127. doi: 10.1073/pnas.1424457112 25775538 10.1073/pnas.1424457112 PMC4386384

[43] Lee DN, Simmons JA, Saillant PA, Bouffard F. Steering by echolocation. A paradigm of ecological acoustics. J Comp Physiol A 1995;176(3): 347–354. 7707270 10.1007/BF00219060

[44] Lee DN, van der Weel FR, Hitchcock T, Matejowsky E, Pettigrew JD. Common principle of guidance by echolocation and vision. J Comp Physiol A 1992;171(5): 563–571. 1494137 10.1007/BF00194105

[45] Kugler K, Greiter W, Luksch H, Firzlaff U, Wiegrebe L. Echo-acoustic flow affects flight in bats. J Exp Biol 2016;219(12): 1793–1797. 27045094 10.1242/jeb.139345

[46] Warnecke M, Lee WJ, Krishnan A, Moss CF. Dynamic Echo Information Guides Flight in the Big Brown Bat. Front Behav Neurosci 2016;10: 81 doi: 10.3389/fnbeh.2016.00081 27199690 10.3389/fnbeh.2016.00081 PMC4843091

[47] Bartenstein SK, Gerstenberg N, Vanderelst D, Peremans H, Firzlaff U. Echo-acoustic flow dynamically modifies the cortical map of target range in bats. Nat Commun 2014;5: 4668 doi: 10.1038/ncomms5668 25131175 10.1038/ncomms5668

[48] Beetz MJ, Hechavarría JC, Kössl M. Cortical neurons of bats respond best to echoes from nearest targets when listening to natural biosonar multi-echo streams. Sci Rep 2016;6: 35991 doi: 10.1038/srep35991 27786252 10.1038/srep35991 PMC5081524

[49] Greiter W, Firzlaff U. Echo-acoustic flow shapes object representation in spatially complex acoustic scenes. J Neurophys 2017;117(6): 2113–2124. doi: 10.1152/jn.00860.2016 28275060 10.1152/jn.00860.2016 PMC5454466

